# Inhibitory Effects of Luteolin 7-Methyl Ether Isolated from *Wikstroemia ganpi* on Tnf-A/Ifn-Γ Mixture-Induced Inflammation in Human Keratinocyte

**DOI:** 10.3390/nu13124387

**Published:** 2021-12-08

**Authors:** Jonghwan Jegal, Tae-Young Kim, No-June Park, Beom-Geun Jo, Geon-A. Jo, Han-Seok Choi, Su-Nam Kim, Min Hye Yang

**Affiliations:** 1 College of Pharmacy, Pusan National University, Busan 46241, Korea; jhjegal@pusan.ac.kr (J.J.); taeyour@pusan.ac.kr (T.-Y.K.); bg_jo@pusan.ac.kr (B.-G.J.); 2 Natural Products Research Institute, Korea Institute of Science and Technology, Gangneung 25451, Korea; 115519@kist.re.kr; 3 Central Research Institute, R&D Strategy, Genuone Sciences, Hwaseong 18449, Korea; geona.jo@genuonesciences.com

**Keywords:** *Wikstroemia ganpi*, atopic dermatitis, tilianin 5-methyl ether, luteolin 7-methyl ether, interleukin 4

## Abstract

Plants of the genus *Wikstroemia* are traditionally used in China to treat various inflammatory diseases. The purpose of this study was to isolate the components of *Wikstroemia ganpi* (Siebold & Zucc.) Maxim., to evaluate their anti-atopic activities and to identify candidates with anti-atopic therapeutics. A total of 24 compounds were isolated by bioassay-guided separation, including one novel compound, which was tilianin 5-methyl ether. The anti-atopic activities of the isolated compounds were determined using TNF-α-treated RBL-2H3 cells and HaCaT cells. The mRNA expressions of IL-4, IL-6, GM-CSF, G-CSF and TRPV1 were reduced by luteolin 7-methyl ether. The study shows that the luteolin 7-methyl ether isolated from *W. ganpi* is a potential therapeutic agent for the treatment of atopic dermatitis.

## 1. Introduction

Atopic dermatitis (AD) is a multifactorial chronic inflammatory skin disease. The most common symptoms of AD are erythema, psoriasis, vesicles, skin tightness and itching accompanied by painful skin lesions [1]. The present study shows that the predominant manifestations of childhood-onset AD include lichenified and/or exudative flexural dermatitis, whereas adult-onset AD s more often characterized by prurigo with highly pruriginous papules and nodules [2]. Itching, combined with scratching, damages the skin barrier, and affected skin reacts sensitively to external allergens, which causes immune defects in inflammatory cells [3]. Immune defects in most AD patients are characterized by upregulating Th2 cytokine and IgE release from mast cells [4]. Representative Th2 cytokines IL-4 and IL-13 suppress filaggrin expression, which plays an important role in the construction of the skin barrier, thereby weakening the function of the skin barrier [5]. IL-31 (Th2-associated cytokine) has also been reported to be an endogenous cause of itching [6], and AD is closely related to pro-inflammatory cytokine (TNF-α, IL-6, GM-CSF, G-CSF and others) released by keratinocytes, dendritic cells, macrophages and other immune cells [3,7]. Furthermore, interactions between pro-inflammatory cytokines and T cell-associated cytokines lead to repeated vicious cycles of itching and chronic inflammatory response [7,8]. To inhibit these inflammatory mediators and reduce itching, topical corticosteroid and topical calcineurin inhibitors are commonly used to treat AD [9]. However, although these AD agents are effective at ameliorating inflammatory immune response and skin conditions, their long-term use may cause side effects [10]. Most recently, crisaborole, a topical phosphodiesterase-4 inhibitor, and dupilumab, an inhibitor of interleukin (IL)-4/13, were FDA-approved, with studies showing an excellent safety profile for chronic treatment in AD [11].

Flavonoids are the major secondary metabolites of plants and have a range of pharmacological effects, which include anti-inflammatory, anti-allergic, and antioxidant effects [12]. These pharmacological properties are attributed to the structural features of flavonoids such as the presence of a C2–C3 double bond, hydroxylation, O-methylation, glycosylation and other substitutions and conjugations [13]. Numerous studies have shown that flavonoids inhibit the expressions of a variety of inflammatory mediators [14,15], and are effective treatments for chronic inflammatory diseases [16]. The inhibition of inflammatory reactions provides a strategy for treating AD [17], and many that investigated the anti-atopic effects of flavonoids and have suggested their potential uses as a natural treatment for AD [18,19,20,21].

*Wikstroemia ganpi* (Siebold & Zucc.) Maxim. is a deciduous shrub and member of the *Wikstroemia* (Thymelaeaceae) genus. It is distributed in Japan, Australia and Korea, and in Korea, is called ‘Geomundodaknamu’ [22]. The plants of the *Wikstroemia* genus are widely used in traditional Chinese medicine to treat a variety of diseases such as syphilis, arthritis and cancer [23]. According to phytochemical reports, members of the *Wikstroemia* genus contain flavonoids, coumarins and lignans, with anti-inflammatory, anti-allergic, antioxidant, anti-viral and other properties [24,25]. However, no study has examined the bioactivity of *W. ganpi*. Recently, we found that *W. ganpi* EtOH extract inhibited inflammatory mediators in DNCB-induced AD mice and attenuated AD-like symptoms [26]. In the present study, we isolated one novel compound, twelve known flavonoids, seven known coumarins, three known lignans, one phenylpropanoid and one phenolic compound from *W. ganpi*, and performed anti-atopic activity screening testing on the isolated bioactive compounds in the hope of identifying a potential anti-atopic agent.

## 2. Materials and Methods

### 2.1. General

NMR (^1^H and ^13^C, HMQC, HMBC and NOESY) spectra were obtained by 500 MHz (Bruker, Billerica, MA, USA) and 600 MHz instruments (Agilent Technologies, Santa Clara, CA, USA). High-resolution ESIMS (HRESIMS) spectra were obtained using an Agilent 6530 Accurate-Mass Q-TOF LC/MS (Agilent Technologies, Santa Clara, CA, USA). HPLC was conducted using a Shimadzu system (Shimadzu Corporation, Kyoto, Japan) equipped with two pumps (LC-20AT), a UV/VIS detector (SPD-20A), and a CBM-20A HPLC system controller. Thin-layer chromatography (TLC) was performed on Merck precoated silica gel 60 F_254_ Art. 5715 (Merck, Germany) plates, and column chromatography was carried out using silica gel (230–400 mesh, Merck, Germany) and Sephadex LH-20 (25–100 mM mesh, Pharmacia, Sweden).

### 2.2. Plant Material and Extraction

The aerial parts of *W. ganpi* (Siebold and Zucc.) Maxim. were collected from Geumsa-ri, Yeongnam-myeon, Goheung-gun, Jeollanam-do, Republic of Korea. Plants were authenticated by Dr. Jin-Hyub Paik (International Biological Material Research Center, Korea Research Institute of Bioscience and Biotechnology), and a voucher specimen (#PNU-0027) was deposited in the College of Pharmacy, Pusan National University. *W. ganpi* samples (~4.27 kg dried plants) were extracted by sonication in 30 L of 95% MeOH for 90 min and left overnight. This process was repeated twice, and the extract obtained was then filtered through Advantec No. 2 filter paper (Advantec, Toyo Roshi Kaish, Ltd., Tokyo). The filtered extracts were concentrated under a vacuum at 35–40 °C using a rotary evaporator, and were then freeze-dried to produce *W. ganpi* MeOH extract (483 g, yield 11.3%).

### 2.3. Isolation of Compounds from W. ganpi Extract

*W. ganpi* MeOH extract was suspended in distilled water and then sequentially partitioned with n-hexane (4 L), EtOAc (4 L) and n-BuOH (4 L). The active EtOAc fraction (41.3 g) was subjected to silica gel CC using CH_2_Cl_2_:MeOH (30:1 → 100% MeOH) as eluent to obtain 17 fractions (WGE1~WGE17). Fraction WGE2 was recrystallized in MeOH to obtain compound **5** (44.5 mg). Fraction WGE3 was recrystallized in MeOH to obtain compound 16 (204.9 mg), and the remaining WGE3 supernatant was subjected to silica gel CC eluted with Hexane:EtOAc (1:1) to yield 15 subfractions (WGE3-1~WGE3-15). Subfraction WGE3-9 was fractionated into 5 fractions (WGE3-9-1~WGE3-9-5) by Sephadex LH-20 (MeOH) CC. Subfraction WGE3-9-2 was subjected to RP HPLC (Watchers 120 ODS-BP, S-10 μm, 150 × 10 mm; detection, UV at 245 nm; flow rate 2 mL/min) by isocratic elution with MeOH-H2O (40:60) to yield compound 13 (5.9 mg, *t*_R_ 27.5 min). Fraction WGE4 was subjected to silica gel CC eluted with CH_2_Cl_2_:MeOH (30:1 → 20:1) to yield 10 subfractions (WGE4-1~WGE4-10). Subfraction WGE4-8 was fractionated into 3 subfractions (WGE4-8-1~WGE4-8-3) by silica gel CC using CH_2_Cl_2_:MeOH (30:1). Subfraction WGE4-8-2 was also subjected to silica gel CC using Hexane:EtOAc (1:1) as eluent to yield 4 subfractions. Subfraction WGE4-8-2-2 was subjected to silica gel CC using Hexane:EtOAc (1:1) to obtain compound **20** (284.7 mg). Compound **21** was obtained by filtering subfraction WGE4-8-3. Subfraction WGE4-9 was recrystallized from MeOH to obtain compound 3 (27.2 mg) and the remaining supernatant was subjected to silica gel CC using Hexane:EtOAc (1:1) as eluent to yield 10 subfractions. Subfraction WGE4-9-2 was subjected to Sephadex LH-20 (MeOH) to obtain compound **24** (11.9 mg). Compound **12** (27.7 mg) was obtained by subjecting subfraction WGE4-9-4 to silica gel CC using Hexane:EtOAc (1:1) as eluent. Fraction WGE 6 was fractionated into 7 subfractions (WGE6-1~WGE6-7) by silica gel CC using Hexane:EtOAc (1:1) as eluent. Subfraction WGE6-7 was subjected to pTLC using (CH_2_Cl_2_:MeOH = 15:1) as eluent to yield compound **22** (14.3 mg, R_f_ = 0.47). Fraction WGE7 was recrystallized from MeOH to obtain compound **4** (310.2 mg) and the remaining supernatant was subjected to silica gel CC using CH_2_Cl_2_:MeOH (20:1 → 10:1) to produce 7 subfractions (WGE7-1~WGE7-7). Subfraction WGE7-6 was subjected to Sephadex LH-20 (MeOH) CC to obtain compound 1 (2.3 mg). Fraction WGE8 was subjected to silica gel CC using CH_2_Cl_2_:MeOH (20:1 → 10:1) to yield 13 subfractions (WGE8-1~WGE8-13). Subfraction WGE8-3 was subjected to Sephadex LH-20 (MeOH) to obtain compound **15** (2.4 mg). Fraction WGE11 was fractionated into 6 subfractions (WGE11-1~WGE11-6) by silica gel CC using CH_2_Cl_2_:MeOH (10:1 → 100% MeOH). Subfraction WGE11-5 was subjected to silica gel CC eluted with CH_2_Cl_2_:MeOH (30:1 → MeOH 100%) to obtain compound **2** (6.8 mg).

The active *n*-BuOH fraction (122.6 g) was subjected to silica gel CC using CH_2_Cl_2_:MeOH (30:1 → 100% MeOH) to yield 9 fractions (WGB1~WGB9). Fraction WGB1 was subjected to silica gel CC using CH_2_Cl_2_:MeOH (20:1 → MeOH 100%) to yield 9 subfractions (WGB1-1~WGB1-9). Subfraction WGB1-4 was subjected to Sephadex LH-20 (MeOH) CC to obtain compound **18** (2.5 mg). Fraction WGB5 was fractionated into 11 subfractions (WGB5-1~WGB5-11) by silica gel CC with CH_2_Cl_2_:MeOH (10:1 → 100% MeOH). Subfraction WGB5-6 was subjected to Sephadex LH-20 (MeOH) CC to yield 4 subfractions. Subfraction WGB5-6-2 was subjected to silica gel CC using CH_2_Cl_2_:MeOH (10:1) to yield 3 subfraction (WGB5-6-2-1~WGB5-6-2-3). Subfraction WGB5-6-2-2 was loaded onto pTLC using (CH_2_Cl_2_:MeOH = 10:1) as eluent to yield compound **14** (2.4 mg, Rf = 0.35). Fraction WGB7 was subjected to silica gel CC eluted using CH_2_Cl_2_:MeOH (10:1) to yield 10 fractions (WGB7-1~WGB7-10). Compound **6** (12.8 mg) was obtained by recrystallizing fraction WGB7-4 from MeOH and fractionating to produce 3 subfractions (WGB7-4-1~WGB7-4-3) by Sephadex LH-20 (MeOH) CC. Subfraction WGB7-4-2 was applied to RP HPLC (Watchers 120 ODS-BP, S-10 μm, 150 × 10 mm; detection, UV at 245 nm; flow rate 2 mL/min) and subjected to isocratic elution using MeOH-H_2_O (60:40) to yield 4 subfractions (WGB7-4-2-1~WGB7-4-2-4). Subfraction WGB7-4-2-2 was subjected to RP HPLC (Watchers 120 ODS-BP, S-10 μm, 150 × 10 mm; detection, UV at 245 nm; flow rate 2 mL/min) to obtain compound **10** (0.9 mg, *t*_R_ 37.6 min). Subfraction WGB7-4-2-4 was identified to compound **11** (1.6 mg, *t*_R_ 31.8 min). Subfractions WGB7-5 and WGB7-6 were recrystallized from MeOH to yield compounds **17** (59.5 mg) and **9** (638.7 mg), respectively. Subfraction WGB7-7 was subjected to silica gel CC eluted with CH_2_Cl_2_:MeOH (10:1) to yield 7 subfraction (WGB7-7-1~WGB7-7-7). Compound **7** was obtained by filtering subfraction WGB7-7-5. Subfraction WGB7-7-7 was subjected to silica gel CC eluted with EtOAc:MeOH (8:1) to yield 4 subfractions (WGB7-7-7-1~WGB7-7-7-4). Subfraction WGB7-7-7-3 was recrystallized from MeOH to obtain compound **19** (1.1 mg). Fraction WGB8 was fractionated into 6 subfractions (WGB8-1~WGB8-6) by silica gel CC using CH_2_Cl_2_:MeOH (10:1 → 100% MeOH). Subfraction WGB8-5 was subjected to silica gel CC eluted with CH_2_Cl_2_:MeOH (20:1 → 100% MeOH) to yield 10 subfractions (WGB8-5-1~WGB8-5-10). Subfraction WGB8-5-8 was subjected to Sephadex LH-20 (MeOH) CC to yield 4 subfractions (WGB8-5-8-1~WGB8-5-8-4). Subfraction WGB8-5-8-1 was loaded onto pTLC and eluted with CH_2_Cl_2_:MeOH (8:1) to obtain compound **23** (3.3 mg, R_f_ = 0.24), and compound **8** (38.0 mg) was obtained by recrystallizing subfraction WGB8-6 from MeOH.

### 2.4. Quantitative Real-Time PCR Analysis of IL-4 mRNA Expression in RBL-2H Cells

RBL-2H3 cells (a rat basophilic leukemia cell-line) were purchased from the Korean Cell Line Bank (Seoul, Korea) and maintained in DMEM (Dulbecco′s Modified Eagle Medium). They were supplemented with 10% FBS (fetal bovine serum; HyClone Laboratories Inc., Logan, UT, USA) and antibiotics (100 U/mL penicillin and 100 μg/mL streptomycin; Invitrogen, Carlsbad, CA, USA) at 37 °C in a 5% humidified CO_2_ atmosphere. Cells were sensitized with DMSO or compounds **1**~**24** at a concentration of 10 μM 1 h and then stimulated with PI (phorbol 12-myristate 13-acetate (PMA) and ionomycin (IOM)) for 9 h. Total RNA was isolated using a RNeasy mini kit (Qiagen, Hilden, Germany). Isolated RNA was reverse transcribed using a RevertAid First Strand cDNA Synthesis Kit (Invitrogen) and IL-4 mRNA levels were assessed by RT-PCR. The sequences of the primers used were as follows: IL-4, forward 5′- ACC TTG CTG TCA CCC TGT TC -3′ and reverse 5′- TTG TGA GCG TGG ACT CAT TC -3′; β-actin, forward 5′-TCA TCA CCA TCG GCA ACG-3′ and reverse 5′-TTC CT GAT GTC CAC GTC GC-3′. Transcript levels were normalized versus β-actin.

### 2.5. Quantitative Real-Time PCR Analysis of the Expressions of TRPA1, TRPV1, IL-31, IL-6, GM-CSF, and G-CSF mRNA in HaCaT Cells

HaCaT cells (an immortalized human keratinocyte cell-line) were provided by professor Seong-Gyu Ko of Kyunghee University. Cells were seeded in 100 mm culture dishes at a density of 1 × 10^6^ cells/mL and incubated for 24 h at 37 ℃, and then treated with 12.5 per second. After real-time quantitative analysis, the results were analyzed using an analysis program. Relative mRNA levels of genes were normalized versus GAPDH mRNA. All experiments were repeated 3 times.

### 2.6. Statistical Analyses

The significances of differences were determined by one-way analysis of variance (ANOVA) and Tukey’s multiple comparisons test. Results are presented as means ± standard errors, and statistical significance was accepted for *p* values < 0.05.

## 3. Results

### 3.1. Isolation of Compounds from W. ganpi Extract and Structural Elucidation of Compound ***11***

**Figure 1:**
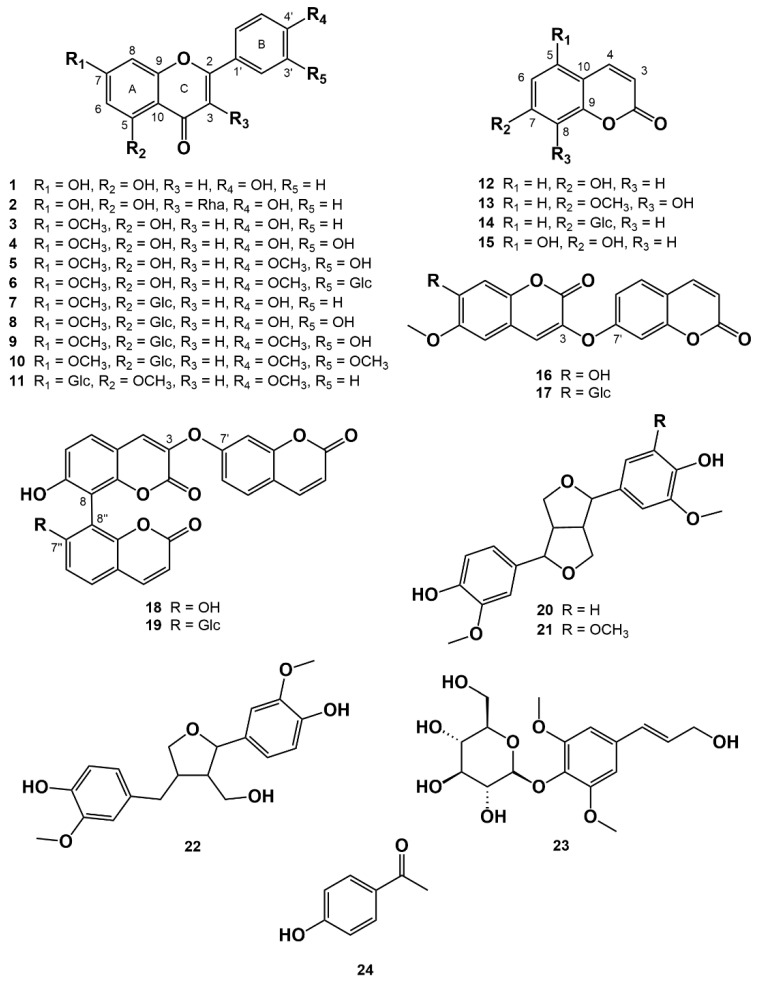
Structures of the compounds isolated from the *W. ganpi* extract.

A one novel flavonoid, ten known flavonoids, eight coumarins, three lignans, one phenylpropanoid and one phenolic compound were isolated from the active EtOAc and *n*-BuOH fractions of *W. ganpi*. The known compounds were identified as apigenin (**1**) [27], afzelin (**2**) [28], genkwanin (**3**) [29], luteolin 7-methyl ether (**4**) [30], pilloin (**5**) [31], pilloin 3′-*O*-glucopyranoside (**6**) [32], genkwanin 5-*O*-glucopyranoside (**7**) [33], yuanhuanin (**8**) [34], pilloin 5-*O*-glucopyranoside (**9**) [31], lethedoside A (**10**) [35] umbeliferone (**12**) [36], daphnetin 7-methyl ether (**13**) [37], skimmin (**14**) [38], 5,7-dihydroxycoumarin (**15**) [39], daphnoretin (**16**) [40], daphnorin (**17**) [41], triumbelletin (**18**) [42], triumbelletin 7′’-*O*-glucopyranoside (**19**) [43], pinoresinol (**20**) [44], medioresinol (**21**) [45], lariciresinol (**22**) [46], syringin (**23**) [47] and 4′-hydroxyacetophenone (**24**) [48], by HREIMS and 1D and 2D NMR (Figure 1).

**Figure 2:**
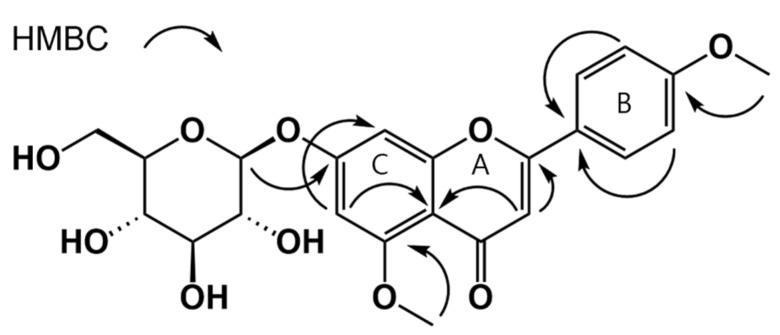
Selected HMBC correlations of tilianin 5-methyl ether (**11**).

Compound **11** was a yellow amorphous powder, and positive-ion HREISMS showed it had a molecular formula of C_22_H_22_O_11_ based on an m/z value of 461.1444. In the ^1^H NMR spectrum, δ_H_ 8.05 (d, *J* = 8.5 Hz, 2H), 7.12 (d, *J* = 8.5 Hz, 2H) peaks suggested 1, 4-substituted aromatic protons of B ring, δ_H_ 7.09 (d, *J* = 2.4 Hz, 1H), 6.91 (d, *J* = 2.4 Hz, 1H) indicated *meta*-coupling of aromatic protons of A ring. Coupled with the above data, the presence of δ_H_ 6.81 (s, 1H) indicated compound **11** was a flavone. The presence of two methoxy was identified by unique peaks (δ_H_ 3.90 (s, 3H), δ_C_ 56.14 / δ_H_ 3.86 (s, 3H), δ_C_ 55.60) in ^1^H and ^13^C NMR spectra. Furthermore, the signal for anomeric protons at 4.77 (d, *J* = 7.6 Hz, 1H) in the ^1^H NMR spectrum suggested a glucopyranoside. HMQC and HMBC spectra analysis led to the full assignment of ^1^H and ^13^C NMR signals (Table 1). The positions of the methoxy and glucopyranosyl groups were determined by correlations of between 5-OCH_3_ protons and C-5, 4′-OCH_3_ protons and C-4′, anomeric proton H-1′’ (glucose) between C-7 respectively in the HMBC spectrum (Figure 2). Based on the data obtained, compound **11** was determined to be tilianin 5-methyl ether.

### 3.2. Screening of Anti-Atopic Activities of Compounds *(**1**–**24**)* Isolated from W. ganpi Extract

**Figure 3:**
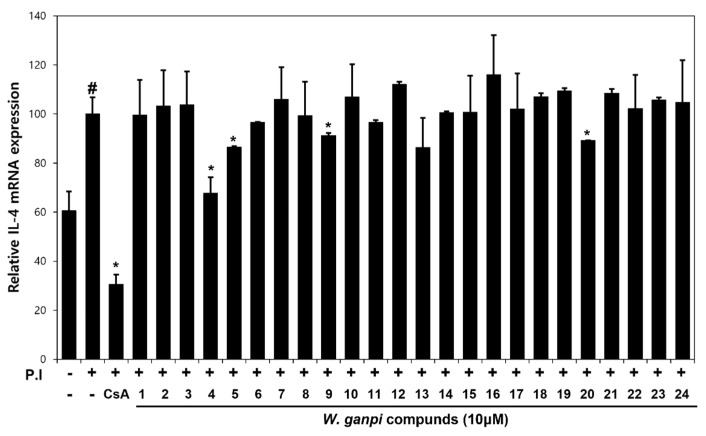
Effects of secondary metabolites isolated from *W. ganpi* on the mRNA expression of the inflammatory cytokine IL-4 expression in PI-induced RBL-2H3 cells. Expression levels of IL-4 mRNA were determined by quantitative real-time PCR relative to β-actin. Data are expressed as the means ± SDs of two independent experiments. *^#^ p* < 0.05 versus vehicle controls. * p < 0.05 vs. the PI-treated cells. PI, PMA/ionomycin; CsA, 10μM cyclosporin; IL-4, interleukin-4.

The inhibitory effects of compounds **1–24** on PI-induced IL-4 mRNA expression were evaluated in RBL-2H3 cells. PI treatment increased the expression of IL-4 mRNA, but treatment with luteolin 7-methyl ether (**4**), pilloin (**5**), pilloin 5-*O*-glucopyranoside (**9**) or pinoresinol (**20**) at 10 μM significantly inhibited this PI-induced increase by about 33%, 14%, 9% and 11%, respectively, as compared with PI-treated controls (ANOVA, *p* < 0.05). In particular, luteolin 7-methyl ether (**4**) pretreatment prevented this PI-induced increase (Figure 3).

### 3.3. Effects of Luteolin 7-Methyl Ether *(**4**)* on the Expressions of IL-6, GM-CSF, and G-CSF mRNA in HaCaT Cells

**Figure 4:**
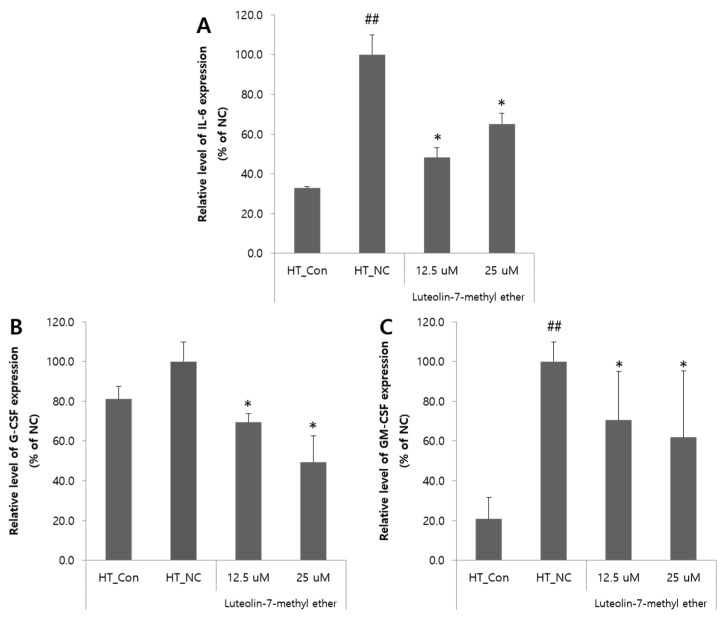
Effects of luteolin 7-methyl ether (**4**) on pro-inflammatory cytokine expressions in TNF-α-treated HaCaT Cells. The expression levels of IL-6 (**A**), G-CSF (**B**), and GM-CSF (**C**) were determined by quantitative real-time PCR relative to GADPH. Data are expressed as the means ± SDs of three independent experiments. ^##^ *p* < 0.01 vs. the vehicle controls. * p < 0.05, vs. TNF-α-treated cells. TNF-α, Tumor necrosis factor-α; G-CSF, Granulocyte colony-stimulating factor; GM-CSF, Granulocyte-macrophage colony-stimulating factor; HT_Con, vehicle control; HT_NC, TNF-α-treated negative control.

To explore the anti-atopic effects of luteolin 7-methyl ether (**4**) further, IL-6, GM-CSF, and G-CSF mRNA expressions were examined in HaCaT cells. Treatment with TNF-α remarkably increased the expression of the pro-inflammatory cytokine IL-6 in the HT_NC, whereas luteolin 7-methyl ether (**4**) treatment reduced IL-6 expression by about 52% at 12.5 μM (ANOVA, *p* < 0.05) (Figure 4A). TNF-α induced increase in G-CSF expression was considerably decreased by luteolin 7-methyl ether (**4**) treatment by 30% at 12.5 μM and 50% at 25 μM, as compared with the HT_NC (ANOVA, *p* < 0.05) (Figure 4B). The treatment with luteolin 7-methyl ether (**4**) also decreased the TNF-α induced increase in GM-CSF expression by 30% at 12.5 μM and 38% at 25 μM (ANOVA, *p* < 0.05) (Figure 4C).

### 3.4. Effects of Luteolin 7-Methyl Ether *(**4**)* on the Pruritus-Related Inflammatory Mediators in HaCaT Cells

**Figure 5:**
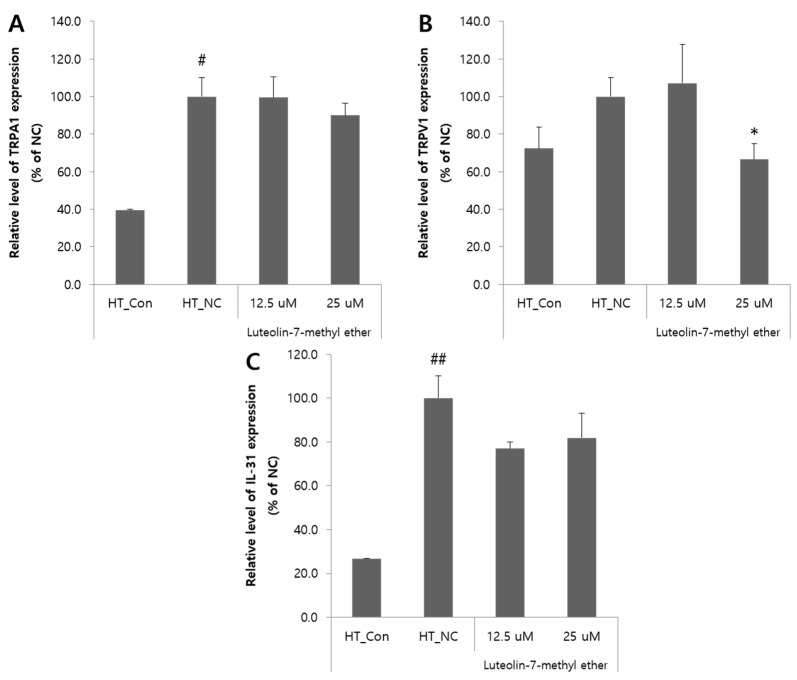
Effect of luteolin 7-methyl ether (**4**) on the expressions of pruritus-related inflammatory mediators in HaCaT cells. The expression levels of TRPA1 (**A**), TRPV1 (**B**) and IL-31 (**C**) were determined by quantitative real-time PCR relative to GADPH. Data are expressed as the means ± SDs of three independent experiments. ^#^ *p* < 0.05 vs. vehicle controls. ^##^ *p* < 0.01 vs. vehicle controls. * p < 0.05, vs. TNF-α-treated cells. TNF-α, Tumor necrosis factor-α; TRPA1, Transient receptor potential ankyrin subtype 1; TRPV1, Transient receptor potential vanilloid 1; HT_Con, vehicle control; HT_NC, TNF-α-treated negative control.

The effects of luteolin 7-methyl ether (**4**) on the mRNA expressions of IL-31 and TRP channel were evaluated. The expressions of TRPA1, TRPV1 and IL-31 mRNA in HaCaT cells were increased by TNF-α treatment and partially decreased by luteolin 7-methyl ether (**4**) treatment. Luteolin 7-methyl ether (**4**) treatment had no significant inhibitory effect on TNF-α-induced TRPA1 overexpression, but at a concentration of 12.5 uM significantly inhibited the overexpression of TRPV1 by about 33% as compared with the HT_NC group (ANOVA, *p* < 0.05) (Figure 5A,B). Luteolin 7-methyl ether (**4**) treatment reduced TNF-α-induced IL-31 mRNA expression, but this was not significant (Figure 5C).

## 4. Discussion

The main clinical symptoms of AD are pruritus, dry skin and lichenification. AD is a common chronic inflammatory skin disease and its prevalence is gradually increasing [7]. AD patients exhibit a characteristic immunological imbalance, in which Th2 cells dominate Th1 cells. IL-4 is a representative cytokine secreted by Th2 cells and plays an important role in the pathogenesis of AD. Elevated IL-4 levels promote antibody isotype switching to IgE, which is involved in inflammatory response [17]. Recently, several studies have indicated that IL-31 is importantly related to atopic dermatitis, and that production of IL-31 is increased by IL-4. Furthermore, in patients with atopic dermatitis, elevated IL-4 and IL-31 levels stimulate sensory nerves and cause itching [6,49]. Topical corticosteroids are being used as first-line treatments to address the immunological imbalance that has profound effects on the induction of AD. However, due to the side effects of topical corticoids, which include skin atrophy and burning and stinging sensations, the long-term use of topical corticoids was prohibited, and thus there is an urgent need for new therapeutic agents.

In a previous study, we investigated the anti-atopic effects of *W. ganpi* in RBL-2H3 mice with DNCB-induced AD, finding that *W. ganpi* EtOH extract (WGE) effectively alleviated AD symptoms and that flavonoids and coumarins are major components of *W. ganpi* using HPLC-PDA [26]. Since the anti-inflammatory and anti-allergic activities of flavonoids have been well demonstrated, it was suggested that the anti-atopic activity of WGE is probably derived from flavonoids [12,15,18]. Based on these studies, we isolated compounds in *W. ganpi* with the aim of identifying a potential therapeutic agent for AD. In total, 11 flavonoids, 8 coumarins, 3 lignans, 1 phenylpropanoid and 1 phenolic compound were isolated using an activity-oriented chromatography, and tilianin 5-methyl ether (**11**) was isolated for the first time. RBL-2H3 cells were each treated with the 24 isolated compounds and then treated with PI – which, when administered alone, induced IL-4 mRNA expression – to determine to what extent degranulation was inhibited. These experiments showed that four compounds, namely, luteolin 7-methyl ether (**4**), pilloin (**5**), pilloin 5-*O*-glucopyranoside (**9**) and pinoresinol (**20**), significantly inhibit PI-induced IL-4 mRNA upregulation.

In addition, we also evaluated the anti-atopic activity of luteolin 7-methyl ether in TNF-α treated HaCaT cells (**4**). TNF-α treatment increased IL-6 expression in HaCaT cells, and this increase was reduced by luteolin 7-methyl ether pretreatment (**4**). IL-6 is involved in cell-mediated inflammation and immune response and enhances B-cell differentiation and T-cell proliferation [50]. Pretreatment with luteolin 7-methyl ether (**4**) also significantly inhibited the TNF-α-induced expressions of G-CSF and GM-CSF in HaCaT cells, and G-CSF and GM-CSF are highly expressed in the keratinocytes of AD patients [51]. To investigate the potential effect of luteolin 7-methyl ether on itching, we examined the expression levels of TRPA1, TRPV1 and IL-31 in HaCaT cells treated with TNF-α. Pretreatment with luteolin 7-methyl ether (**4**) was found to significantly decrease TNF-α-induced TRPV1 expression, and to slightly decrease the TNF-α-induced expressions of TRPA1 and IL-31.

To summarize, 24 compounds were isolated by activity-guided bioassay from the methanol extract of the aerial parts of *W. ganpi,* and these compounds included the flavonoid tilianin 5-methyl ether (**4**), which is reported here for the first time. When RBL-2H3 cells were pretreated with the isolated compounds and then treated with TNF-α, luteolin 7-methyl ether (**4**) was found to significantly inhibit the expression of IL-4, which is known to be closely related to AD. In addition, luteolin 7-methyl ether (**4**) also inhibited the expressions of cytokines and ion channels involved in inflammation and pruritus in TNF-α-induced HaCaT cells. Our experimental results suggest that luteolin 7-methyl ether (**4**), the main active ingredient of WGE, has potential as a treatment for AD.

## Figures and Tables

**Table 1:** ^1^H NMR (DMSO, 500 MHz) and ^13^C NMR (DMSO, 126 MHz) NMR spectral data of compound **11** in DMSO-*d_6._*

	Compound 11	
Position	δ_H_ (*J* in Hz)	δ_C_
2		161.02
3	6.81 (s)	106.53
4		177.01
5	6.91 (d, 2.4)	163.67
6		103.46
7		158.26
8	7.09 (d, 2.4)	96.61
9		158.55
10		109.31
1′		122.78
2′, 6′	8.05 (d, 8.5)	128.11
3′, 5′	7.12 (d, 8.5)	114.61
4′		162.15
Glc 1	4.77 (d, 7.6)	104.10
2	3.52–3.43	73.62
3	3.45–3.37	75.80
4	3.19–3.12	69.98
5	3.52–3.43	77.68
6	3.78–3.70 3.19–3.12	60.97
5-OCH_3_	3.90	56.14
4′-OCH_3_	3.86	55.60

## Data Availability

The data presented in this study are available on request from the corresponding author.
